# Expression of clec9a in the oral cancer microenvironment. A preliminary immunohistochemical pilot study

**DOI:** 10.4317/medoral.24659

**Published:** 2021-08-19

**Authors:** Juan Francisco Peña-Cardelles, José Juan Pozo-Kreilinger, Giovanna Roncador, Jesús Esteban-Hernández, José Luis Cebrián-Carretero, José Ernesto Moro-Rodríguez

**Affiliations:** 1 PhD Student, Health Sciences Faculty, Universidad Rey Juan Carlos, Madrid, Spain; 2 MD, DDS, PhD. Associate Professor of Medicine. Department of Pathology. Universidad Autoìnoma de Madrid, Hospital Universitario La Paz, Madrid; 3 BSc, PhD. Head of the Monoclonal Antibody Unit, Spanish National Cancer Research Center (CNIO), Madrid, Spain; 4 BSc, PhD. Associate Professor. Public Health and Preventive Medicine Unit. Health Sciences Faculty, Universidad Rey Juan Carlos, Madrid, Spain; 5 MD, DDS, PhD. Head of Section, Department of Oral and Maxillofacial Surgery, Hospital Universitario La Paz, Madrid; 6 MD, PhD. University Professor. Pathological Anatomy Area, Universidad Rey Juan Carlos, Madrid, Spain

## Abstract

**Background:**

The search for treatments to improve cancer survival has led to the emergence of immunotherapy and the study of the tumour microenvironment existing in neoplasms. This preliminary study aims to understand the clinical and pathological relationship of clec9a expression in oral cancer and to explore survival models for future studies.

Material and methods: Immunohistochemical study that included 26 patients with a diagnosis of oral squamous cell carcinoma (OSCC) in mobile tongue and floor of the mouth. Clinical and histopathological variables were recorded, and the biomarkers clec9a for dendritic cells and CD8 and CD4 for lymphocytes were used.

**Results:**

Clec9a was expressed in 58% of the sample. It was more common in cases with low lymphoplasmacytic infiltration and in type 2 invasion patterns. It was significantly related to CD8 expression (*p*=0.055 and *p*=0.007). No prognostic risks were evident in the survival models studied (overall survival, disease-specific survival, disease-free survival).

**Conclusions:**

CLEC9A expression is present in the OSCC microenvironment and is mainly related to the presence of CD8 lymphocytes. The relationship of its expression with survival prognosis in OSCC could not be confirmed; however, this needs to be confirmed through future studies with larger sample size.

** Key words:**Clec9a, dendritic cells, tumor microenvironment, oral cancer, immunotherapy.

## Introduction

The latest GLOBOCAN shows that a total of 354,864 people were diagnosed with oral squamous cell carcinoma (OSCC) in 2018, and 177,384 people died of oral cancer, a trend that is increasing (1). The search for treatments to improve cancer survival has led to the emergence of immunotherapy and a greater understanding of the tumour microenvironment (TME) in neoplasms.

The role that different cells can play in the TME is beginning to be discerned (2). However, no previous research has studied the role of clec9a+ dendritic cells (DC) in oral cancer, and this research is the first to do so.

Clec9a corresponds to a molecule on the surface of CD141+ DCs that can interact with necrotic cell proteins to process and to present to immune cells (3).

This preliminary study aims to gain insight into the clinical and pathological relationship of clec9a expression in oral cancer and to explore survival patterns for future studies.

## Material and Methods

A retrospective observational epidemiological study that included 26 patients with a diagnosis of OSCC in mobile tongue and floor of the mouth location who were examined at the Oral and Maxillofacial Surgery Department and the Anatomical Pathology Department of La Paz University Hospital (HULP) in Madrid between 2010 and 2014.

- Collection of clinical data

The study included patients with an anatomopathological diagnosis of primary OSCC after surgical resection in the anterior tongue (C02.0, C02.01) and/or floor of the mouth (C04) in the time interval from 2010 to 2014.

Exclusion criteria: 1. Patients who had been treated before the surgical removal of the tumour with oncological therapy, whether pharmacological or radiotherapeutic. 2. Cases with a diagnosis before 2010 or after 2014. 3. Patients with a positive diagnosis for the human immunodeficiency virus. 4. Cases whose diagnosis was oral carcinoma with micro invasion. 5. Cases in which relevant information was missing and with insufficient histological material to be able to perform histopathological analysis.

The clinical variables of sex, age, smoking habit (never, current or former smoker), alcoholic habit (never, current or former alcoholic habit), the primary location of the tumour, the dates of diagnosis and treatment (surgery, radiotherapy and chemotherapy), the presence of relapses (local, regional and distant) and the presence of oral potentially malignant disorders (OPMDs) were collected based on the latest classification of these lesions (4).

The characteristics of the neoplasm such as tumour size, the presence of regional or distant metastases were registered according to the latest TNM classification of the head and neck region of the American Joint Committee on Cancer (AJCC) (5).

- Preliminary anatomopathological analysis and selection of histological blocks

Tissue samples were provided by the Pathology Department of the HULP. All samples were analysed under optical microscopy simultaneously by three independent observers, two doctors specialised in Anatomic Pathology with a subspecialty in oral pathology, as well as a dentist specialised in Oral Medical Pathology and knowledgeable in the histopathological analysis of the oral anatomical region. Before the analysis of the samples, a study and consensus on how to determine the histological features for each sample were carried out by the three observers to establish a comparison in the determination of these features.

Based on the histopathological characteristics, the most representative paraffin blocks were selected for each case, first evaluating the diagnostic biopsy and then selecting the block from the surgical specimen of the tumour with a macroscopic observation and then a microscopic observation, confirming that it was an objective and representative sample of the tumour.

Histological features were studied, including host lymphoplasmacytic response (HLPR), the worst pattern of invasion (WPOI) and perineural invasion (PI); features collected according to the criteria of the histological risk model definitions.

Furthermore, the histological grade of the tumour was recorded, classified as poor (PD), moderate (MD) or well-differentiated (WD) as well as whether the tumour had vascular and/or lymphatic invasion (6).

The depth of invasion (DOI) was recorded. This was done by first determining whether the lesion was exophytic or ulcerated, then by drawing a horizontal line delimiting the basal membrane and a vertical line ("plumb line") from the basal membrane to the invasion front of the tumour, classifying them as minor invasive lesions (≤ 5 mm), moderate (> 5 mm and ≤ 10 mm) and deep invasive lesions (> 10 mm). After this registry, the TNM classification of the tumours was updated, as it may be modified according to the values obtained in the DOI (7).

- Immunohistochemistry

Immunohistochemical staining was performed as follows: 2-μm-thick sections were prepared from formalin-fixed paraffin-embedded tissue blocks and were dried in a 60°C oven overnight. The sections were placed in a Bond Max Automated Immunohistochemistry Vision Biosystem (Leica Microsystems GmbH, Wetzlar, Germany) according to the following protocol. First, tissues were deparaffinized and pre-treated with the Epitope Retrieval Solution 2 (EDTA-buffer pH8.8) at 100°C for 20 min. After wash steps, peroxidase blocking was carried out for 10 min using the Bond Polymer Refine Detection Kit DC9800 (Leica Microsystems GmbH). Tissues were again washed and then incubated with the primary antibodies for 30 min. Subsequently, tissues were incubated with polymer for 15 min and then with DAB-Chromogen for 10 min.

**Figure 1:**
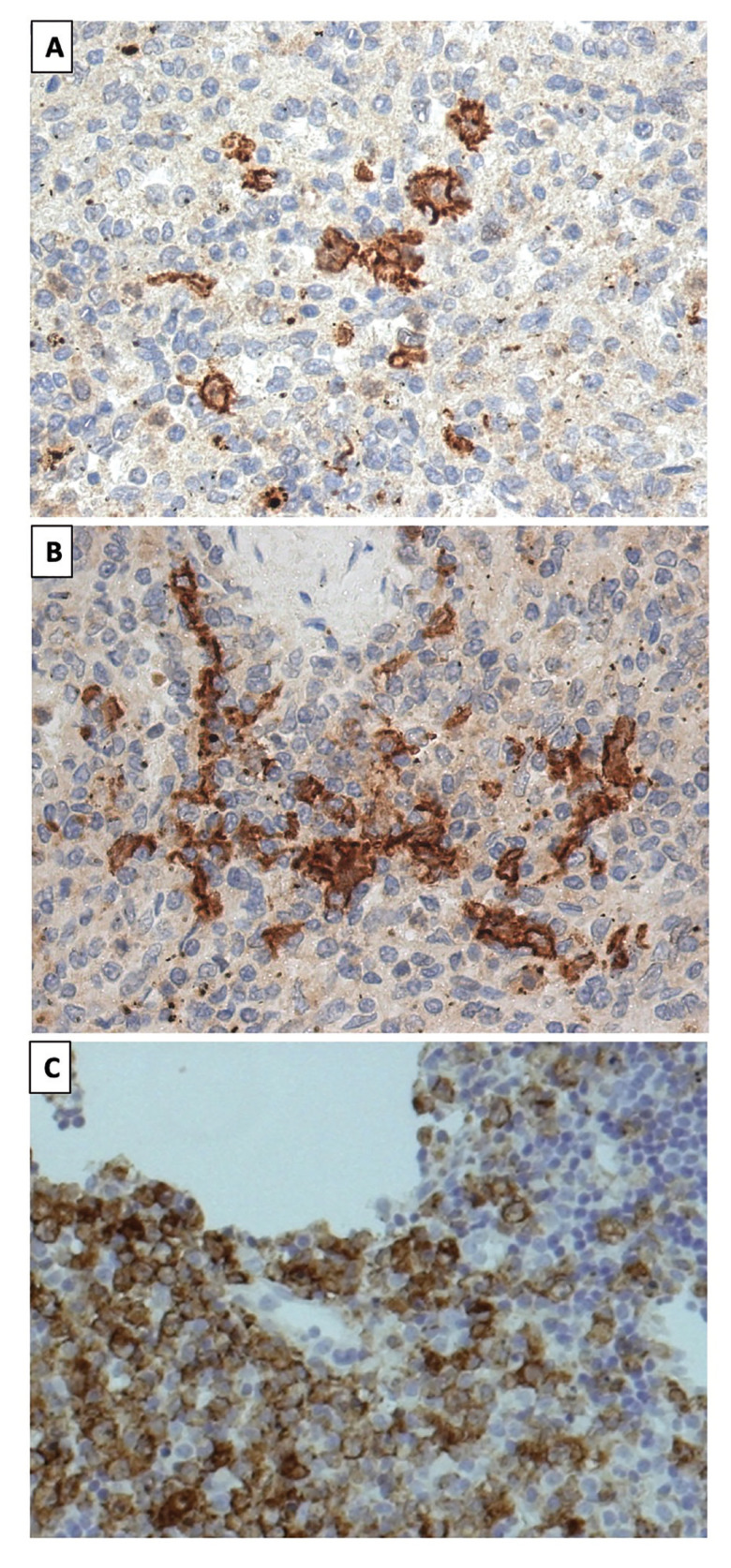
Microphotographs of different Clec9a positive human tonsil controls at 20x magnification.

Positive and negative human tonsil controls were run in parallel. Incubations either omitting the specific antibody or containing unrelated antibodies were used as a control of the technique (Fig. 1).

Primary antibodies were used following this technique: CLEC9A (Antibody type: Rat monoclonal, clone number: LEIA256A, dilution: prediluted, source: CNIO), CD4 (Antibody type: Mouse monoclonal, clone name: 4B12, dilution: prediluted, source: DAKO) CD8 (Antibody type: Rat monoclonal, clone name: NOR132H, dilution: 1:5 supernatant, source: CNIO) and p16 biomarker (antibody type: mouse monoclonal; clone name: E6H4; source: Roche).

- Immunohistochemical interpretation

The Immunohistochemical interpretation was performed between the three observers who analysed the histopathological features of the samples by simultaneous co-observation. The assessment of the three biomarkers, on the one hand, CLEC9A was performed at the membrane and cytoplasm level, on the other hand, CD4 and CD8 was performed at the membrane level. The percentage expression of stained cells was calculated as a percentage of the total number of cells in the sample and the cells were counted manually.

**Figure 2:**
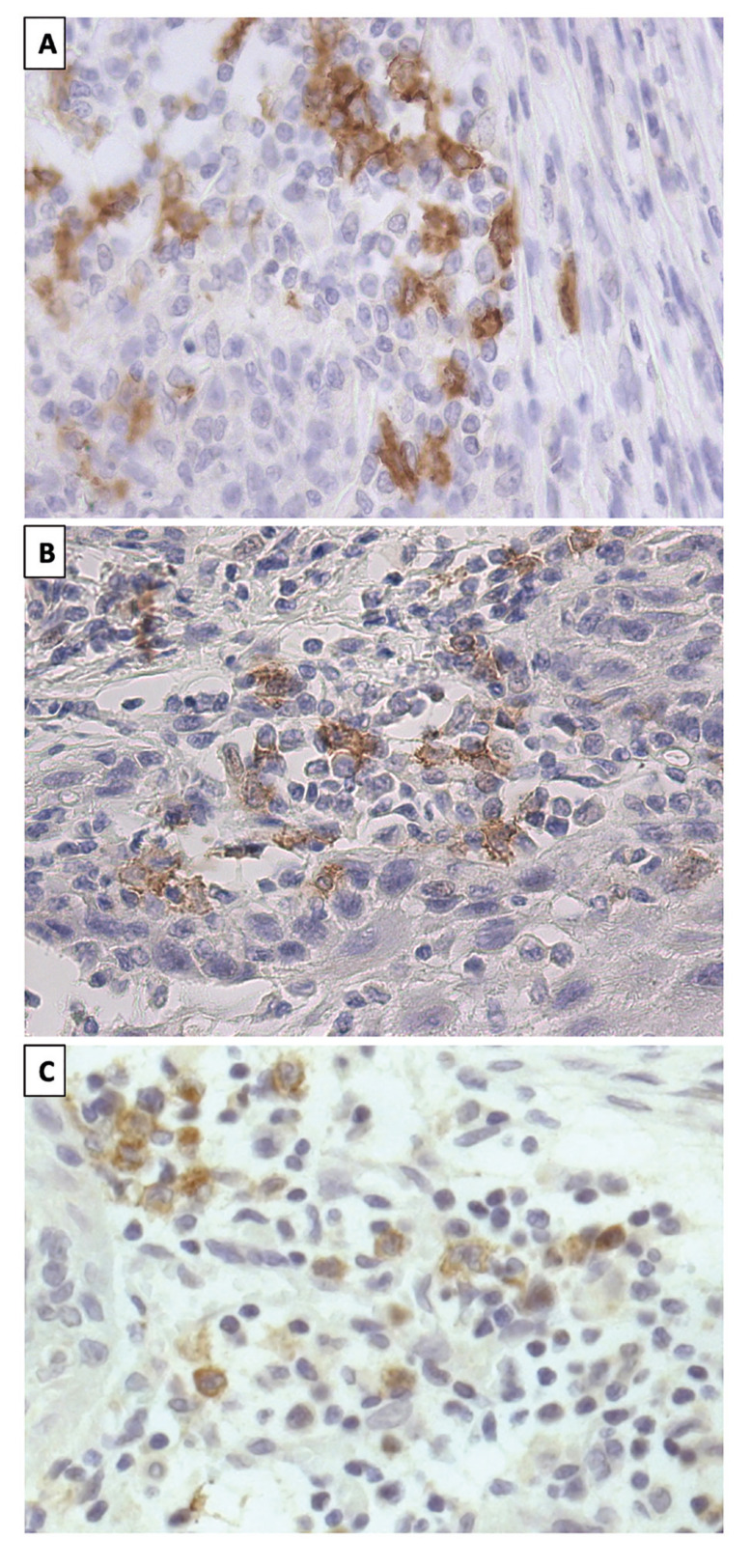
Microphotograph of immunohistochemistry of clec9a in OSCC. A. Image by light microscopy (20×) of histological sections of a sample whose total expression of Clec9a was 9% (Positive, expression > 0%). B. Microphotograph showing 3% Clec9a expression (Positive, expression > 0%) at 20× magnification. C. 20× image of the clec9a biomarker in a sample with a total expression of 5% (Positive, expression > 0%).

For an objective collection of biomarker expression, samples were observed under light microscopy at 10x, 20x and 40x magnification and the percentage of biomarker expression was calculated (Fig. 2).

The biomarkers were categorized for statistical study as follows.

CLEC9A. Cases with expression >0% were classified as positive and cases <0% as negative. CD8 (expression 0-10% (minor), 10-50% (moderate), ≥50% (severe). CD4 in three groups: 5-25%, 25-35% and 35-50%.

- Survival

Survival data were collected by analysing patient records. The time from the date of diagnosis to the outcome of interest was measured to the nearest month. The definition of outcomes was defined as follows: Oral cancer death, death from another cause, recurrence (regardless of whether local, regional or distant) and alive without recurrence. Based on these outcomes, three definitions were considered: Disease-specific survival (DSS), where only death from oral cancer was considered as an event; Disease-free survival (DFS), where recurrence (of any type) or death from oral cancer (but not death from another cause) was considered as an event; and Overall survival (OS), where events were defined as death from any cause.

- Statistical analysis

Continuous variables were described by mean and standard deviation when their distribution was normal and median and interquartile range when their distribution was not normal. Discrete variables were presented as counts and percentages. Differences in continuous variables were tested by one-way ANOVA when the assumptions of normality and homoscedasticity were met, and Kruskal-Wallis when not. Where necessary, pairwise comparisons were obtained after ANOVA or Kruskal-Wallis using Tukey-Kramer and Dunn's tests, respectively. Pearson's chi-square test or Fisher's exact test, as appropriate, helped to test for homogeneity between proportions.

Kaplan-Meier survival functions were plotted using the same cut-off point and compared using the Wilcoxon-Breslow-Gehan test.

Survival analysis was performed with the RMS package (version 6.0-1) and Kaplan-Meier curves were plotted with the survminer package (version: 0.4.8). All analyses were performed with R (v 4.0.3, GNU GPL-3) and RStudio (version 1.3.959, GNU GPL-3).

## Results

- Sample selection 

The initial sample consisted of 36 cases of patients with a primary diagnosis of OSCC in the floor of the mouth and mobile tongue after consulting the database of the Oral Surgery Department between 2010 and 2014.

From the initial sample to the final selection, 10 cases were excluded (4 non-OSCC neoplasms, 1 case with insufficient histological material for the study of histopathological features, 3 cases of carcinoma with microinvasion and 2 cases of OSCC with locations other than the floor of the mouth and anterior lingual region), making a total of 26 cases to be studied in this research.

- Clinical and histopathological characteristics

The clinical and histopathological characteristics of the patient sample are shown in Table 1.

The study finally included 14 males (54%) and 12 females (46%) with an average age of 66 years (interquartile range of 61 and 76). 65% had never smoked, with 15% being former smokers and 19% current smokers. The most frequent primary location was the tongue (92%) and in the case of the floor of the mouth, it was 7.7%. None of the cases occurred in both locations simultaneously. 65% of the tumours were stage III or IV. 19% of the patients (n=5) had OPMDs before the diagnosis of OSCC.

Histopathologically, 58% of the tumours were moderately differentiated, the evaluation of the worst invasive pattern (WPOI) included 62% of the cases in type 2. Perineural invasion was present in 35% of patients and vascular invasion in 3.8%. Lymphoplasmacytic infiltration was mostly low in 58% of cases.

The median DOI was 8 mm. 42% of patients were minor (≤5 mm), 27% moderate (6-10 mm) and 31% deep (≥10 mm).

To assess the positivity of human papillomavirus (HPV) through p16, the criteria of the College of American Pathologists were used. Positive cases were considered those whose tumour tissue showed nuclear and cytoplasmic immunoreactivity in ≥ 70% of the cells (8). Following this criteria no p16 positive case was found.

- Clec9a expression

Clec9a was expressed in 58% of the sample, corresponding to 15 cases versus 11 negative cases (42%), being slightly higher in females than in males (*p*=0.134). Cases with smaller tumour size (T1+T2) were more positive (*p*=0.683). Regarding stage, there was a higher expression in stage III cases (*p*=0.702).

CLEC9A positivity was associated with cases with low lymphoplasmacytic expression (*p*=0.124) and concerning WPOI, the vast majority of positive cases were WPOI 2 (*p*=0.492).

The mean CD8 expression was 25%, while CD4 expression was 20%. The expression of clec9a was significantly associated with CD8 expression (*p*=0.055) and with a strong association with the expression of the moderate CD8 group (*p*=0.007). However, no such relationship existed with CD4 expression.

Table 2 shows the distribution of different clinical and histopathological variables according to CLEC9A positivity.

**Table 1:**
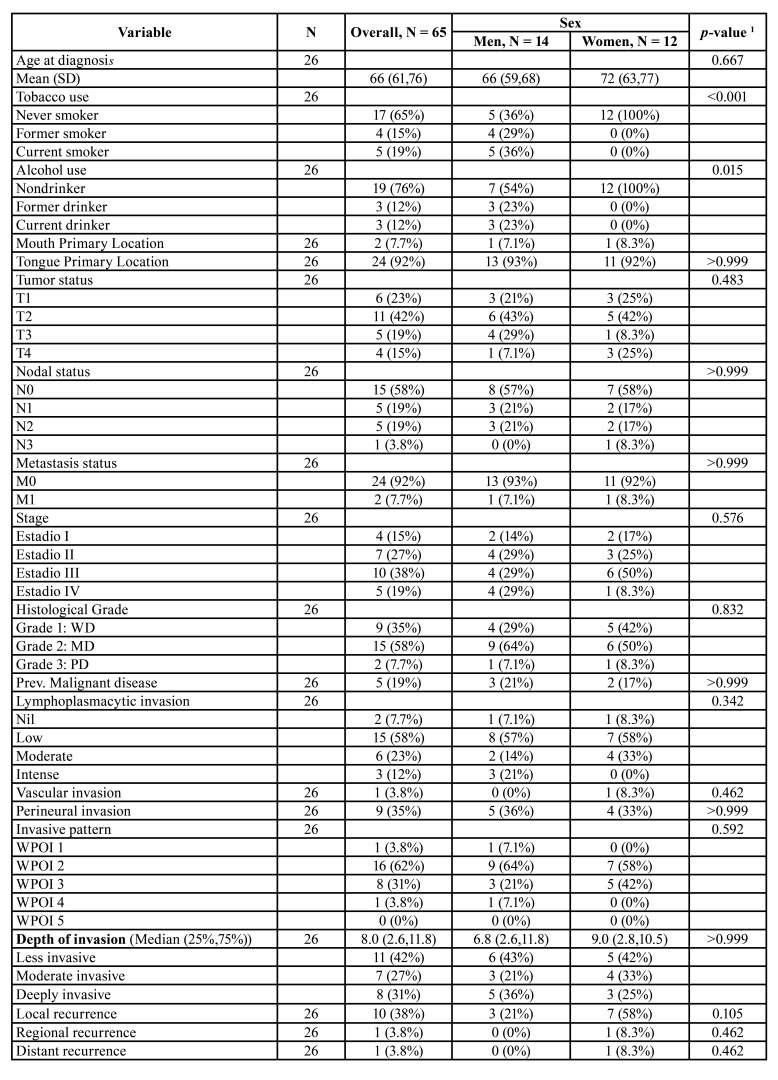
Descriptive statistics of clinical and histopathological variables according to sex.

**Table 1 cont.**
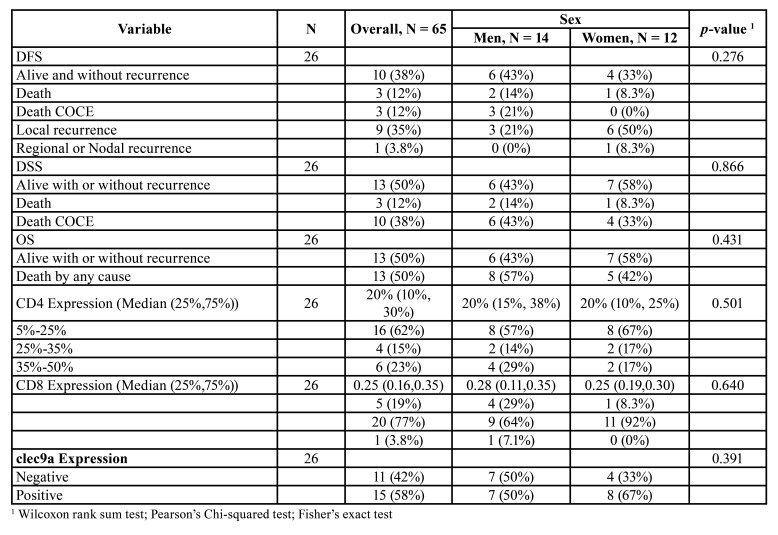
Descriptive statistics of clinical and histopathological variables according to sex.

**Table 2:**
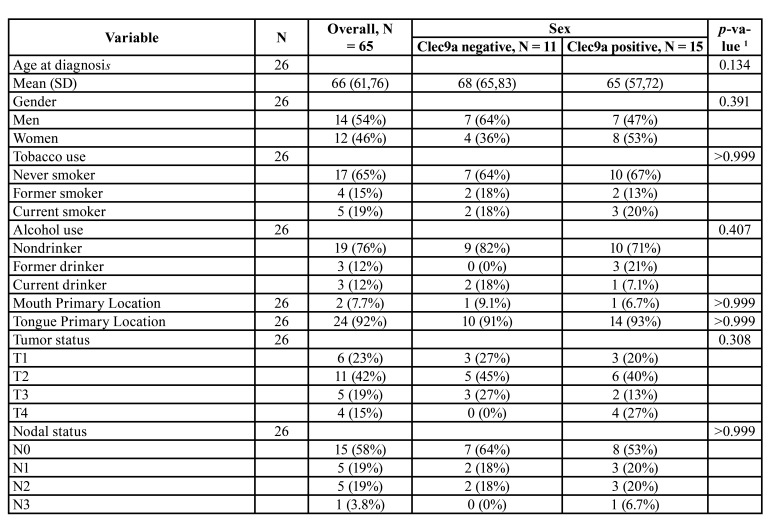
Descriptive statistics of clinical and histopathological variables according to expression of clec9a.

**Table 2 cont.**
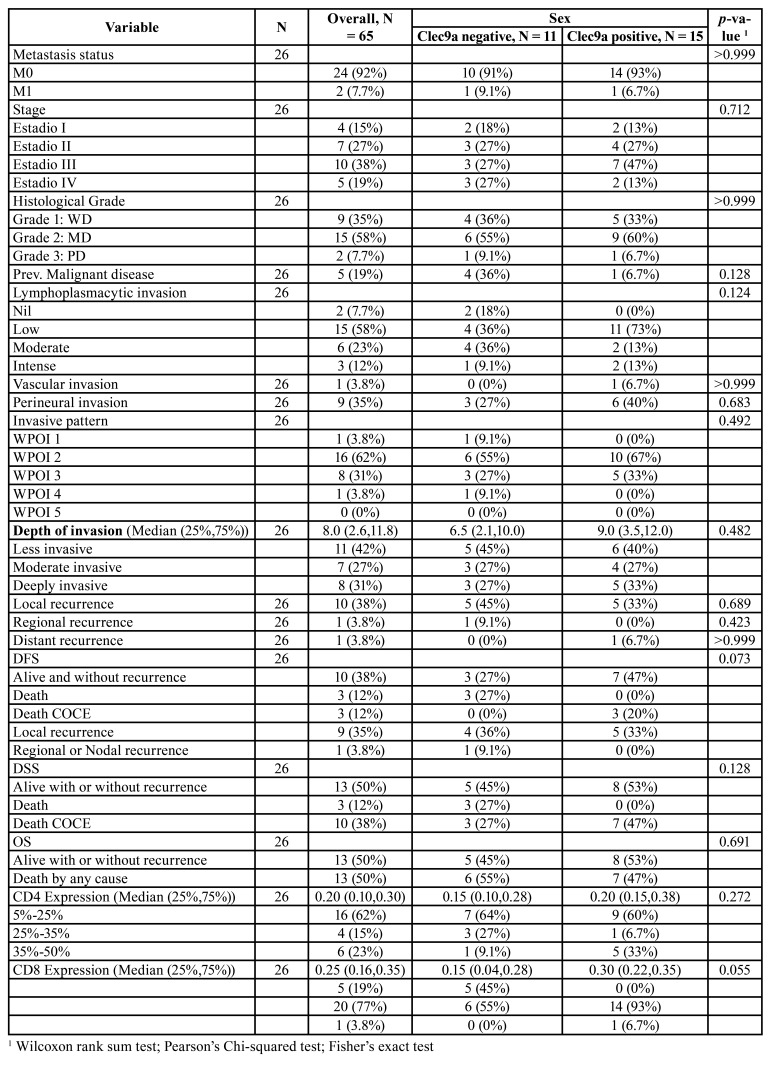
Descriptive statistics of clinical and histopathological variables according to expression of clec9a.

- Survival analysis

During a median follow-up (OS) of 83 months (p25-p75: 55-105), 13 deaths (50%) by any cause (38% by OSCC) were observed. There were no losses to follow-up.

As for the comparison of event proportion as a function of clec9a positivity in survival, no strong association was evidenced (OS, *p*=0.7, DSS *p*=0.4, DFS *p*=0.7) (Table 3).

**Figure 3:**
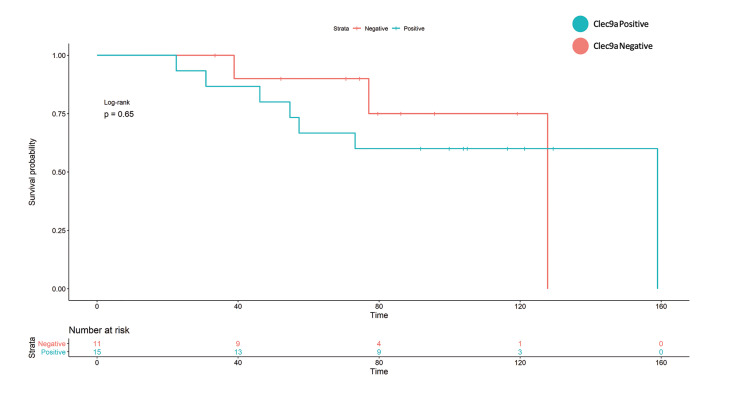
Kaplan Meier analysis of the DSS model. It can be observed that despite the lack of events and therefore lack of statistical power, there may be a difference in survival.

Kaplan-Meier analysis does not show an increased hazard for OS, nor DFS, however, in the case of DSS, there appears to be a small difference in survival (Fig. 3).

## Discussion

The present preliminary study is the first investigation to study the presence of Clec9a+ DCSs in the TME of oral cancer. The findings show that they are present in the OSCC TME and that they are significantly related to the presence of CD8 T lymphocytes.

**Figure 4:**
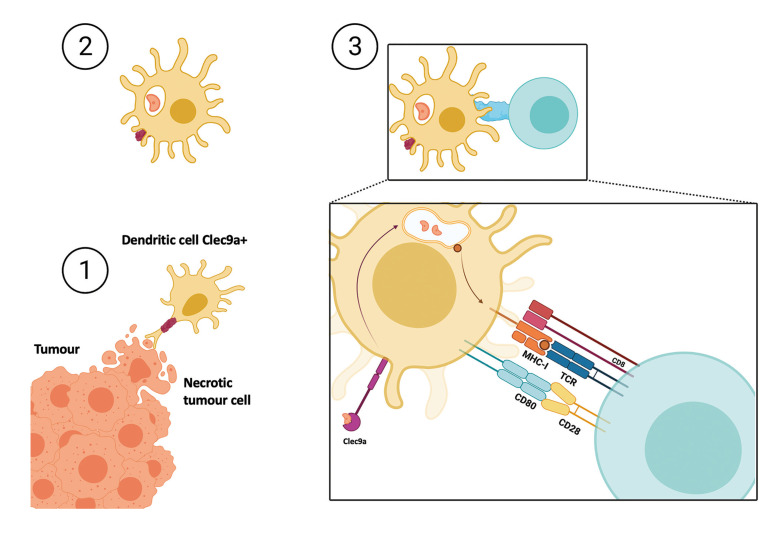
Representative image of the steps of a DC identifying a antigen present in necrotic tumor cell. Step 1. Clec9a+ dendritic cell interacting with a necrotic tumour cell. Step 2. Through clec9a, the dendritic cell has taken up the tumour antigen and processed it at the endosomal level for further presentation. Step 3. Presentation of the necrotic antigen to the CD8 T-lymphocyte via the type I histocompatibility complex.

CD8 T lymphocytes are important cells in the immune response to the tumour. It is the clec9a positive DCs that can present necrotic antigens to CD8 lymphocytes via the type I histocompatibility complex (Fig. 4) (9,10).

**Table 3:**
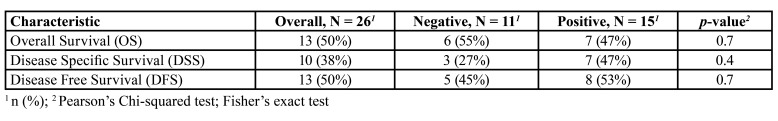
Comparison of survival rate according to positivity for clec9a.

Clec9a corresponds to a molecule on the surface of CD141+ DCs. It is an actin filament that can interact with necrotic cell proteins to process them later (3,11). They may therefore play an important role in the immune system's response to cancer. This fact has led to the current study of anti-tumour vaccines using these cells in immunotherapy (11-14). Such studies appear to show positive results in animal models (15,16).

Previous studies in mouse models have shown that the loss of precursors for this type of DCs increases the poor prognosis in breast and pancreatic cancer (17). Likewise, other studies in ovarian cancer indicate that the functional lack of 141+ DCs precursors is associated with a worse prognosis (18) and a study highlights that the administration of 141+ Clec9a+ DC stimulates CD8 T lymphocytes in Willis tumour and concludes that it is a promising candidate of immunotherapy in malignant neoplasms (19).

It appears from the findings of this study that the presence of these clec9a+ DCs is important in the role of tumour-infiltrating CD8 lymphocytes (TILS), as both are expressed at the same time. The role of TILS in the TME has been linked in previous studies to a better prognosis for survival, however, other cells in the microenvironment such as macrophages or regulatory T cells may favour disease progression (20).

To know for sure the role of CLEC9A+ DCs, we believe that a study with a larger sample size should be carried out to explore statistical contrasts that could not be detected in the preliminary research, as well as the possibility of comparing CLC9A expression in oral cancer to non-neoplastic inflammatory tissue. As shown in Table 3, no relationship has been found between clec9a expression and survival in this disease. Furthermore, we believe that a study with a larger sample size will allow a multivariate analysis to discern the role of CLEC9A and CD8 separately.

Histologically, clec9a+ expression has been positive with low degrees of lymphoplasmacytic infiltration, as well as in WPOI type 2. This type of WPOI is defined by some authors as having a low risk of tumour recurrence (21). However, low lymphoplasmacytic infiltration has been associated in previous studies with worse prognosis due to higher recurrences (22).

It should be taken into consideration that the sample of this study presents the aetiological factors of tobacco and alcohol with a low prevalence (65% non-smokers and 76% non-drinkers), although this should be confirmed with a larger sample size, the study of HPV aetiology was carried out and all cases were p16 negative by immunohistochemistry, ruling out this possible aetiological factor. Recent studies indicate that oral cancer and potentially malignant oral lesions that occur in patients without the classical aetiological factors of tobacco and alcohol have a similar genetic basis (23,24). On the other hand, the gender distribution of the sample is very similar (54% men and 42% women), matching the similar distribution in recent studies even though the male sex has always had a higher prevalence (25).

Therefore, the initial results of this pilot study indicate that the prognostic role of clec9a should be explored as it may have implications for the TME and immunotherapy.

## Conclusions

CLEC9A expression is present in the OSCC microenvironment and is mainly related to the presence of CD8 lymphocytes.

The relationship of its expression with survival prognosis in OSCC could not be confirmed, however, this needs to be confirmed by future studies of larger sample size.
